# Commentary: Parkinson disease-linked GBA mutation effects reversed by molecular chaperones in human cell and fly models

**DOI:** 10.3389/fnins.2016.00578

**Published:** 2016-12-23

**Authors:** Hugo J. R. Fernandes, Brent J. Ryan, Richard Wade-Martins

**Affiliations:** ^1^Institute of Cancer and Genomic Sciences, College of Medical and Dental Sciences, University of BirminghamBirmingham, UK; ^2^Department of Physiology, Anatomy and Genetics, Oxford Parkinson's Disease Centre, University of OxfordOxford, UK

**Keywords:** ER stress, Parkinson's disease, ER stress response, dopaminergic neurons, Drosophila, GBA, chaperones, therapeutics

A recent thorough study by Sanchez-Martinez et al. (2016) highlighted the pathological role of ER stress in Parkinson's disease (PD), finding ER stress associated with dopaminergic cell death and PD-like locomotor deficits in a *Drosophila* GBA-PD model. Importantly, the authors observed that motor impairments could be rescued by two pharmacological chaperones that reduced ER stress, highlighting a route for potential therapeutic intervention.

The endoplasmic reticulum (ER) is a central organelle for protein folding, lipid synthesis, and calcium storage. Disturbances in ER homeostasis can be caused by multiple factors, including accumulation of misfolded proteins, oxidative stress, and calcium imbalance, resulting in ER stress. This leads to the activation of the unfolded protein response (UPR) triggering a cascade of events aimed at restoring ER homeostasis, or under severe stress activating cell death pathways (Walter and Ron, 2011).

Emerging evidence supports a pathogenic role for ER stress across many common neurodegenerative diseases, including PD, Alzheimer's disease, amyotrophic lateral sclerosis, and Huntington's disease (reviewed in Scheper and Hoozemans, 2015). In these conditions, ER stress has been mostly associated with the accumulation of misfolded proteins, a common characteristic in neurodegeneration. In PD, ER stress has been confirmed in a variety of models, including post-mortem brain tissue from sporadic disease (Hoozemans et al., 2007), toxin models of PD (Ryu et al., 2002; Holtz and O'Malley, 2003), a yeast alpha-synucleopathy model (Cooper et al., 2006) and A53T mouse and rat PD models (Colla et al., 2012). More recently, we and others also confirmed increased ER stress in iPSC-derived neuronal models from PD patients carrying GBA mutations (Fernandes et al., 2016) and α-synuclein (*SNCA*) mutations and triplications (Chung et al., 2013). Taken together, these and other reports suggest a central role for ER stress in PD but the precise mechanisms leading to pathogenesis are still unclear.

Sanchez-Martinez et al. demonstrated that the expression of human mutant forms of GCase, commonly associated with PD—N370S and L444P—was abundant in the ER, resulting in the formation of ER aggregates and swellings associated with increased ER stress. Strikingly, both mutations resulted in an age-dependent and progressive climbing defect in *Drosophila*, resembling a PD motor-like symptom. Moreover, this was also associated with a loss of dopaminergic neurons, without affecting life-span or inducing wide-spread degeneration. This time-dependent and cell-specific dysregulation is an important aspect of disease modeling for age-dependent neurodegenerative diseases such as PD.

By treating N370S and L444P mutant flies with two pharmacological chaperones which are known to reduce ER stress the authors confirmed a link to the described motor deficits. Treatment with ambroxol or isofagomine strongly reduced ER stress and significantly improved the climbing deficits caused by human mutant GBA. However, the potential association between the recovery observed and improved dopaminergic neuron survival was not investigated. Whilst isofagomine treatment also significantly increased GCase activity and protein levels in L444P flies, no improvements were observed in N370S flies, in contrast to the results from patient fibroblasts. Similarly, ambroxol rescue effects on GCase activity and protein levels were modest in flies with either the N370S or L444P mutation, but striking in fibroblasts. Additional work is therefore required to elucidate the relative contributions of GCase activity and GBA trafficking to mutant GBA-induced dysfunction, which might be cell type specific and genotype dependent.

Overall, although this study highlights a pathological role for ER stress in PD, it should be also noted that in the fly model, WT GBA overexpression alone induced ER stress, which might have not been induced at physiological levels of expression. This highlights the importance of confirming these results in future studies using patient iPSC-derived neurons complemented with gene editing strategies.

Importantly, these results come in strong agreement with other recent *Drosophila* studies where human *GBA*-N370S and *GBA*-L444P expression also resulted in ER stress, leading to dopaminergic cell loss and climbing deficits which could be rescued by ambroxol (Maor et al., 2013, 2016). The success of another ER stress reducing agent (salubrinal) in delaying or attenuating motor abnormalities in *SNCA*-A53T mouse and rat models of PD (Colla et al., 2012) further highlights the therapeutic potential of this approach.

As several reports suggest, ER stress is a pathological PD feature not limited to GBA mutations, and the mechanisms supporting other genetic association are now emerging. Very recently, Celardo et al. (2016) showed that in *Drosophila pink1* and *parkin* PD models, defective mitochondria lead to ER stress and proposed a neuroprotective mechanism driven by inhibition of ER stress that was independent of mitochondrial functional improvement. In addition, α-synuclein has been shown to modulate UPR and ER stress response by blocking ATF6 incorporation into COPII vesicles (Credle et al., 2015). While several other PD genes, including LRRK2, DJ-1, and ATP13A2 have also been associated with ER stress (reviewed in Mercado et al., 2013), further mechanistic studies are still needed to better understand this association.

Notably, most cellular pathways that have been associated with PD are directly regulated by the ER, including calcium homeostasis, ER-to-Golgi trafficking, protein folding, autophagy, mitochondria-associated ER membranes (MAM), oxidative stress, and lipid metabolism. Together with key pathological PD features such as α-synuclein aggregation and mitochondrial dysfunction, most of these pathways have been associated with ER stress across different models. For some of these processes however, further work is still required in the specific context of PD, for which we would highlight the emerging role of lipid metabolism and the dynamics of MAM in the interplay between ER stress, mitochondrial function, and autophagy. Finally, since ER stress is associated with aging and known to regulate longevity, further studies on the effect of aging on ER stress in the context PD would be welcomed, as supported by the age-dependent neurodegeneration phenotypes presented by Sanchez-Martinez et al. (2016).

To conclude, the work by Sanchez-Martinez et al. (2016) supports previous studies in providing strong and complementary evidence to support a key pathological role of ER stress across multiple models of PD, which can potentially be treated therapeutically. Nevertheless, further studies are still required to better understand the molecular pathways underlying ER stress across the different mutations and cellular pathways associated with PD.

## Author contributions

HJRF: conceived and wrote the commentary; BJR and RW-M: review and critique. All authors approved it for publication.

## Funding

Work at the Oxford Parkinson's Disease Centre is supported by the Monument Trust Discovery Award from Parkinson's UK (Grant ref: J-1403).

### Conflict of interest statement

The authors declare that the research was conducted in the absence of any commercial or financial relationships that could be construed as a potential conflict of interest.
